# Retraction Note: Autophagy-independent induction of LC3B through oxidative stress reveals its non-canonical role in anoikis of ovarian cancer cells

**DOI:** 10.1038/s41419-024-07025-6

**Published:** 2024-09-04

**Authors:** Eswara Murali Satyavarapu, Ranjita Das, Chandan Mandal, Asima Mukhopadhyay, Chitra Mandal

**Affiliations:** 1 Cancer Biology and Inflammatory Disorder Division, Council of Scientific and Industrial Research-Indian Institute of Chemical Biology, 4, Raja S.C. Mallick Road, Kolkata, 700032 India; 2 Bose Institute, P 1/12, C. I. T. Road, Scheme – VIIM, Kolkata, 700054 India; 3 Tata Medical Center, 14 MAR, Rajarhat, Kolkata, 700156 India; 4 Northern Institute for Cancer Research, Newcastle University, Newcastle, UK

Retraction Note to: *Cell Death and Disease* 10.1038/s41419-018-0989-8, published online 17 September 2018

The Editors have retracted this article because there appear to be anomalies in three of the figures. Specifically

- Duplication of GADPH bands between Figs. 1d and 4g.
- Partial overlap of the first and fourth panels in Fig. 3i.
- Repeated regions in the third panel of Fig. 2e.
- Duplication of the Atg12 band in Fig. 2i with the beta-actin band in Fig. 4d.
- Duplication of the Bax band in Fig. 3n with the Slug band in Fig. 4g after flipping horizontally.

The Editors, therefore, no longer have confidence in the results and conclusions presented. Eswara Murali Satyavarapu, Ranjita Das, Chandan Mandal, and Chitra Mandal have not responded to correspondence from the Publisher about this retraction. The Publisher was not able to find a current email address for Asima Mukhodhyay.

